# Biological parameters predictive of percent dense red blood cell decrease under hydroxyurea

**DOI:** 10.1186/s13023-015-0272-3

**Published:** 2015-05-09

**Authors:** Marie Georgine Rakotoson, Gaetana Di Liberto, Etienne Audureau, Anoosha Habibi, Christine Fauroux, Sanam Khorgami, Anne Hulin, Sylvain Loric, France Noizat-Pirenne, Frédéric Galacteros, Pablo Bartolucci

**Affiliations:** Institut Mondor de Recherche Biomédicale, Unité 955, Equipe 2: Transfusion et Maladies du Globule Rouge, Université Paris-Est Créteil, Créteil, France; Etablissement Français du Sang, Île-de-France Mondor, Créteil, France; Service de Santé Publique, Hôpital Henri-Mondor, APHP, LIC EA4393, Université Paris-Est Créteil, Créteil, France; Centre de Référence des Syndromes Drépanocytaires Majeurs, Hôpital Henri-Mondor, APHP, Université Paris-Est Créteil, 51, avenue du Mal-de-Lattre-de-Tassigny, 94010 Créteil Cedex, France; Laboratoire de Pharmacologie, APHP, Hôpital Henri-Mondor, Université Paris Est-Créteil, Créteil, France; Laboratoire de Biochimie et Génétique, Hôpital Henri-Mondor, Créteil, France

**Keywords:** Sickle-cell–disease, Dense red blood cells, Hydroxyurea

## Abstract

**Background:**

Dense red blood cells (DRBCs) are associated with chronic clinical manifestations of sickle-cell–disease (SCD). Hydroxyurea (HU) decreases the percent (%) DRBCs, thereby improving its therapeutic benefits, especially the prevention of SCD clinical complications, but parameters influencing %DRBCs remain unknown. The purpose of this study was to determine predictive biological parameters of %DRBC decline under HU.

**Methods:**

Factors affecting the %DRBC decrease in SCD patients HU-treated for ≥6 months were analyzed. Biological parameters and the %DRBCs were determined before starting HU and after ≥6 months of HU intake. Bivariate analyses evaluated the impact of each biological parameter variation on %DRBC changes under treatment. Multivariate analyses assessed the correlations between the decreased %DRBCs and biological parameters.

**Results:**

The %DRBCs declined by 40.95% after ≥6 months on HU. That decrease was associated with less hemolysis, however in several analyses on this group of patients we did not find a statistically significant correlation between decrease in %DRBCs and increase in HbF. Initial %DRBC values were the most relevant parameter to predict %DRBC decline.

**Conclusion:**

Our results strengthen the known HU efficacy in SCD management statistically independently of the classical HbF biological response. Decreasing %DRBCs is essential to limiting chronic SCD symptoms related to DRBCs and predictive factors might help prevent those manifestations. The results of this study provide new perspectives on indication for HU use, i.e., to prevent SCD-induced organ damage.

## Background

Dense red blood cells (DRBCs) are a subpopulation of RBCs intricately involved in SCD clinical manifestations and are defined as having a density >1.11 mg/mL [1,2]. They are characterized by a higher mean corpuscular hemoglobin concentration (MCHC) [3-7], because of dehydration caused by K^+^ loss. Dehydration also promotes hemoglobin S (HbS) polymerization, depending on the 20^th^–40^th^ power of the HbS concentration [8]. The percent DRBCs remains stable (no differences for 26 patients with 3.2 ± 1.8 years of follow-up, p = 0.79) [9], and is a biological characteristic of homozygous SCD patients at steady state not taking hydroxyurea (HU). DRBCs play an important role in SCD pathophysiology because of their hemorheological properties and their hemolytic propensity. The %DRBCs is strongly associated with chronic vasculopathy manifestations, e.g., renal dysfunction, leg ulcers, or priapism [9].

HU effectively limits SCD vaso-occlusive crisis, acute chest-syndrome frequencies and mortality. The classical biological parameter of the response to HU is increased fetal hemoglobin (HbF), a potent anti-HbS–polymerization factor. However, the results of some studies showed that HU can obtain biological benefit independently of increasing HbF, by inhibiting a membrane protein responsible for cell adhesion [2,10].

Accumulating evidence strengthens the HU indication for chronic vasculopathy, which is associated with the %DRBCs. We previously showed that HU lowers the %DRBCs after 6 months, but information is lacking on the biological determinants of a good response, in terms of fewer %DRBCs, that could support treatment onset for a not yet common indication. Herein, we demonstrate that the strongest predictive factor of HU efficacy is the baseline %DRBCs itself, underpinning its expected good efficacy in patients at high risk of chronic vasculopathy.

## Methods

### Patient characteristics and study design

This prospective, longitudinal, monocenter study included 56 SCD patients taking HU regularly and followed in our Adult Sickle-Cell–Disease Referral Center. Patients >18 years old with SS SCD proven by Hb electrophoresis were eligible for inclusion. Non-inclusion criteria were pregnancy, blood transfusion during the previous 3 months and refused consent. Patients taking HU were monitored for 6–12 months (≥M6). The local Institutional Review Board (CPP–Île-de-France IV Saint-Louis Hospital) approved this study. In accordance with the Declaration of Helsinki, all patients gave their signed informed consent; all data were rendered anonymous to protect patients’ privacy and confidentiality.

### Biological parameter measurements

Blood was drawn at steady state during outpatient visits. We collected biological parameter values before treatment (day 0, D0) and after ≥ M6 on HU. MCHC (g/dL), mean corpuscular Hb content (MCH; pg), Hb (g/dL), mean corpuscular cell volume (MCV; fl), white blood-cell (WBC; G/L), reticulocyte (G/L) and platelet counts (G/L) were determined with a Coulter LH 750 counter (Beckman Coulter, Villepinte, France).

RBC-density curves were obtained with the phthalate density-distribution technique [11]. Serum levels of total bilirubin (μmol/L), lactate dehydrogenase (LDH; IU/L), alanine aminotransferase (ALT; IU/L) and aspartate aminotransferase (AST; IU/L) were assessed with a chemical analyzer (Advia 1650; Siemens Medical Solutions Diagnostics, Holliston, MA, USA).

The %HbF was determined by high-pressure liquid chromatography of Hb using the Variant II Hemoglobin Testing System (Bio-Rad Laboratories, Marnes-la-Coquette, France).

### Statistical analyses

Descriptive results are expressed as means ± standard deviation (SD) or percentages. Bivariate analyses used the paired *t*-test or Wilcoxon signed-rank test for comparisons of continuous biological parameters between D0 and ≥ M6, as appropriate. Pearson correlation coefficients were computed to assess the relationship between %DRBC decline and collected biological parameters, considering first their D0 to M6 variation and then %DRBC decline compared to the pretreatment values. Baseline parameters associated with %DRBC change achieving p < 0.2 in unadjusted bivariate analyses were entered into multivariate linear-regression models to identify independent predictors of %DRBC decrease under HU. The coefficient of determination (r^2^) was used to estimate the percent variability explained by the model. A two-tailed p < 0.05 defined significance. All statistical analyses were computed with Stata software v12.1 (StataCorp LP, College Station, TX, USA).

## Results

### Patients

Fifty-six SS patients (24 men and 32 women; mean age 33.7 ± 9.5 years) were included in this study. HU was prescribed for the following indications: acute chest syndrome (31), repeated vaso-occlusive crises (21), severe anemia (2), overt stroke (1) or pulmonary hypertension (1). The mean HU dose was 15 mg/kg/day taken in a single oral dose.

A larger %DRBC decrease over the ≥6 months was correlated with aging (r = −0.332; p = 0.013). No significant relationship was found between sex and the %DRBC change. The %DRBCs had decreased significantly from 10.5 ± 8.7% to 6.2 ± 4.5% (p = 0.0003). All biological parameter values varied between D0 and after ≥ M6 on HU (Table 1).

**Table 1:** **Before and after ≥ M6 HU and bivariate analyses of biological parameter variations and %DRBC change**

**Parameter**	**Biological parameter values (mean ± SD)**			**Bivariate analysis**	
	**Before**	**After ≥ M6**	**p value**	**r**	**p value**
**%DRBCs**	10.48 ± 8.75	6.18 ± 4.49	0.0003		
**%HbF**	7.17 ± 4.13	17.45 ± 8.14	<0.0001	−0.080	0.556
**Hb (g/dL)**	9.04 ± 1.28	9.91 ± 1.67	<0.0001	−0.071	0.602
**WBCs (G/L)**	10.09 ± 2.54	7.10 ± 2.03	<0.0001	0.200	0.140
**MCV (fl)**	89.16 ± 8.04	108.69 ± 12.74	<0.0001	−0.059	0.665
**MCHC (g/dL)**	33.44 ± 1.23	33.48 ± 0.84	0.796	−0.022	0.874
**MCH (pg)**	29.84 ± 3.23	36.43 ± 4.64	<0.0001	−0.069	0.615
**Reticulocytes (G/L)**	244.92 ± 80.84	146.48 ± 64.99	<0.0001	0.238	0.077
**Platelets (G/L)**	396.41 ± 149.51	325.49 ± 106.09	0.0002	−0.069	0.614
**Total bilirubin (μmol/L)**	45.96 ± 26.76	35.64 ± 20.48	<0.0001	**0.335**	**0.012**
**ALT (IU/L)**	29.81 ± 16.22	26.89 ± 12.14	0.212	0.068	0.620
**AST (IU/L)**	44.04 ± 17.13	39.67 ± 16.26	0.149	**0.311**	**0.020**
**LDH (IU/L)**	433.02 ± 180.19	368.98 ± 139.66	0.0004	**0.363**	**0.006**

Numerical data in bold indicate significant correlation with %DRBC decrease.

### %DRBC decrease was associated with reduced hemolysis markers but not HbF variation

**Figure 1:**
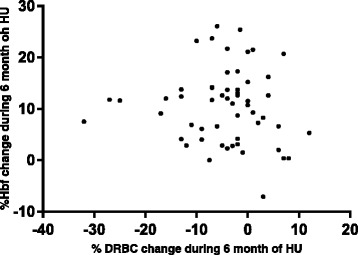
Correlations between change in %HbF and change in %DRBCs (r = −0.080; p = 0.556).

Modifications of monitored biological parameters and %DRBC change during therapy were analyzed. Bivariate analyses (Table 1) showed that the %DRBC diminution was positively and significantly correlated with reductions of total bilirubin, AST and LDH (p = 0.012, 0.020 and 0.006, respectively). Expressing unadjusted linear-regression results as β-coefficients, declines of one total bilirubin, AST or LDH unit were correlated, respectively, with 0.118, 0.113 or 0.019 %DRBC diminutions. Pertinently, in this group of patients we did not find a statistically significant correlation between decrease in % DRBCs and increase in HbF (r = −0.080; p = 0.556) (figure 1) or MCV variation (r = −0.059; p = 0.665) throughout HU treatment. To reinforce our analysis of HbF variation according to the %DRBC decrease, %DRBC change was tested in each %HbF-change quartile that comprised 14 patients. Again, no between-group differences were observed according to the %DRBC decline (p = 0.32).

### Baseline parameters predictive of %DRBC diminution under HU

**Figure 2:**
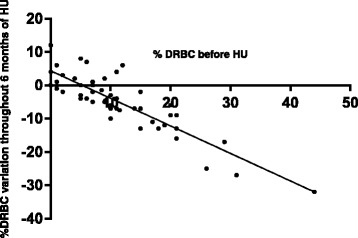
Correlations between %DRBC variation and D0 %DRBCs (r = −0.885, p < 0.0001).

Correlations between D0 biological parameters and %DRBC decline were also evaluated (Table 2). Multivariate analyses retained the D0 %DRBCs as the only significant predictor of %DRBC decline at ≥6 months (p < 0.0001). Patients with high D0 %DRBCs had the largest %DRBC decreases (Figure 2). This statistical model explained 76.8% of the %DRBC diminution. No correlation was found between %DRBC reduction and D0 Hb, HbF or MCV (p = 0.501, 0.484 and 0.902, respectively). Furthermore, HbF variation under HU was not statistically correlated with D0 HbF (p = 0.847).

**Table 2:** **Bivariate and multivariate analyses of D0 biological parameters associated with %DRBC change under HU**

	**Bivariate analysis**		Multivariate analysis*	
**Parameter**	**r**	**p value**	**β-coefficient**	**p value**
**%DRBCs**	**−0.863**	**<0.0001**	**−0.885**	**<0.0001**
**WBCs (G/L)**	−0.257	0.068	–	–
**MCV (fl)**	−0.018	0.902	–	–
**MCHC (g/dL)**	**−0.485**	**0.0004**	0.668	0.404
**MCH (pg)**	−0.140	0.332	–	–
**Hb (g/dL)**	0.097	0.501	–	–
**%HbF**	0.099	0.484	–	–
**Reticulocytes (G/L)**	**−0.437**	**0.002**	−0.004	0.703
**Platelets (G/L)**	0.200	0.159	–	–
**Total bilirubin (μmol/L)**	**−0.426**	**0.002**	0.034	0.285
**ALT (IU/L)**	−0.144	0.310	–	–
**AST (IU/L)**	**−0.419**	**0.002**	−0.023	0.285
**LDH (IU/L)**	**−0.324**	**0.020**	−0.001	0.801

*Model also adjusted for age and WBC count: r^2^ = 0.768.

Numerical data in bold indicate significant correlation with %DRBC decrease.

## Discussion

This study was undertaken to identify predictors of %DRBC decrease in a cohort of 56 SS SCD patients taking HU. The %DRBCs dropped by 40.95% after ≥6 months on HU. We previously reported a 34% %DRBC decrease [9]. HU diminished SCD patients’ DRBC levels, thereby leading to fewer organ complications and, thus, clinical improvement [2,5,12,13].

Our results demonstrated that the parameter best predicting the %DRBC diminution was the D0 %DRBCs itself, with a negative correlation: the higher the D0 %DRBCs, the greater the %DRBC decline. This marked %DRBC decrease further supports HU efficacy on biological and clinical SCD features.

Analyzing the possible relationship between %DRBC diminution and the evolution of each biological parameter, results showed strong associations between %DRBC decline and hemolysis parameters. Total bilirubin, AST and LDH decreased with %DRBC, in agreement with previously reported observations that HU lowers the levels of hemolysis markers [12,14].

While our previous results showed a correlation between %DRBC and HbF values in a cohort study on untreated patients [9], in several analyses on this group of patients we did not find a statistically significant correlation between decrease in % DRBCs and increase in HbF in terms of therapeutic effects. The lack of correlation between %DRBC decrease and HbF increase was confirmed by determining %DRBC change according to HbF-variation quartiles. Charache et al. studied 32 HU-treated patients [13] and did not find a correlation between HbF increase and %DRBC decrease or hemolysis parameters. These striking observations clearly indicate that HbF is one of the mechanisms but may not be the main pathway through which HU reduces the %DRBCs during the course of treatment. Moreover, Goldberg et al. showed that the HbF plateau was reached after 6 months whereas DRBCs were rapidly removed from the circulation according to a biphasic erythrocyte-survival curve [12]. That observation supports the findings that some biological effects of HU on RBC adhesion [10], ionic transport or MCV [12,15] are faster than HbF evolution, or could be totally independent, as for endothelial cells [16]. The process underlying the elimination or decrease in %DRBCs under HU has not yet been completely elucidated. KCl cotransport, Na^+^ pumps and Ca^2+^-dependent K^+^-efflux (Gardos) channels are involved in RBC dehydration and, thus, in DRBC formation [17]. HU has been shown to increase RBC [2,12,18] and endothelial cell K^+^ contents [16], in part by reducing the KCl-cotransport rate, thereby facilitating RBC hydration. According to those observations, HU presumably acts, but not exclusively, by raising HbF; however, HU might also modify RBC-hydration properties involved in SCD pathophysiology.

Notably, we did not find any correlation between the D0 HbF level and its increase, in accordance with a previous study [19].

Because DRBCs and hemolysis are clearly associated with some chronic organ dysfunction that markedly contributes to SCD morbidity and mortality [9,20], prescribing HU early could be an option to prevent those events in patients with very high %DRBCs, which is one of the best markers of chronic organ involvements. Indeed, authors of recent studies argued for a preventive role of HU in the appearance of organ damage [21,22].

## Conclusion

Our findings confirmed HU efficacy at decreasing the %DRBCs, which was associated with less hemolysis, and that D0 %DRBC values were the most relevant parameter predictive of SCD progression. These observations strongly support that, even in the absence of a HbF response, HU could obtain clinical improvement.

The %DRBC decrease, a simple and reproducible biological marker of hemolysis and chronic vasculopathy, could serve as a target for treatment management and clinical SCD trials.

The HU mechanism of action on %DRBC decline remains unclear, since it has only rarely been studied [9,18]. To fully understand this unclear point, longitudinal and kinetic analyses of the %DRBC decrease throughout HU therapy would be required to complete our results and further investigations are also needed to explain the role of HU in RBC-rehydration pathways.

## Abbreviations

ALT: Alanine aminotransferase
AST: Aspartate aminotransferase
DRBCs: Dense red blood cells
Hb: Hemoglobin
HbF: Fetal hemoglobin
HbS: Hemoglobin S
HU: Hydroxyurea
LDH: Lactate dehydrogenase
MCH: Mean corpuscular Hb content
MCHC: Mean corpuscular hemoglobin concentration
MCV: Mean corpuscular cell volume
RBCs: Red blood cells
SCD: Sickle-cell–disease
WBCs: White blood cells
